# Epstein-Barr Virus Infection-associated Hemophagocytic Lymphohistiocytosis

**DOI:** 10.7759/cureus.7563

**Published:** 2020-04-06

**Authors:** Pamela Contreras-Chavez, Andrea Anampa-Guzmán, Safwan Gaznabi, Frederick Lansigan

**Affiliations:** 1 Internal Medicine, Advocate Illinois Masonic Medical Center, Chicago, USA; 2 Internal Medicine, Universidad Nacional Mayor De San Marcos, Lima, PER; 3 Hematology, Dartmouth-Hitchcock Norris Cotton Cancer Center, Lebanon, USA

**Keywords:** epstein-barr virus infection, haemophagocytic syndrome, epstein-barr virus

## Abstract

Hemophagocytic lymphohistiocytosis (HLH) is a rare and life-threatening syndrome characterized by uncontrolled immune activation. There is an aberrant activation of lym­phocytes and macrophages that results in hypercytokinemia. We aim to describe a case of secondary HLH due to primary Epstein-Barr virus (EBV) infection. A Hispanic 28-year-old man presented with sore throat and fatigue for one week. He was diagnosed with mononucleosis and discharged and was treated according to the currently available treatment. HLH is treated by diminishing the inflammation by myelosuppressive and immunosuppressive therapy. EBV infection-associated HLH is a rare disease with high mortality. It is crucial to think about it when facing a patient with fever, cytopenia, hepatosplenomegaly, and high levels of ferritin. Despite medical treatment, the patient died from multiorgan failure.

## Introduction

Hemophagocytic lymph histiocytosis (HLH) is a deadly, unregulated activation of macrophages and lym­phocytes that results in a cytokine storm [1]. HLH is a heterogeneous syndrome and can be divided into primary or secondary.

Primary HLH is caused by mutations affecting proteins involved in the trafficking and exocytosis of cytotoxic granules in cytotoxic T cells and natural killer cells. It can also be caused by mutation of PRF1, which encodes the cytotoxic protein perforin itself and additional mutations in UNC13D (also known as MUNC13-4), STX11, or STXBP2 [2]. Affected individuals have this predisposing genetic defect in immune function and present HLH by age one year often after infection [3]. On the other hand; secondary HLH occurs mostly in adults with acquired defects in cytotoxic T lymphocytes and natural killer cell function. It is reactive and can be triggered by infection, autoimmune disease, and malignancy [4].

Diagnostic criteria of HLH were established at the Histiocyte Society HLH 2004 [5]. The patient must present with either or both of the following: documented molecular conformation consistent with HLH and five or more of the following findings: fever, splenomegaly, cytopenias affecting multiple lineages in peripheral, hypertriglyceridemia and or hypofibrinogenemia, hemophagocytosis in bone marrow, spleen, or lymph nodes; low or absent natural killer cell activity; and high ferritin levels and high soluble CD25 level [6]. The most common presentation is fever, cytopenia, and hepatosplenomegaly. In later phases, most patients develop multiorgan failure [7]. It is important to diagnose it early because HLH has a mortality rate of 41% from a retrospective study of 1109 adults [8].

## Case presentation

A Hispanic 28-year-old man presented to the emergency department (ED) with sore throat and fatigue for one week. He was diagnosed with Epstein-Barr Virus (EBV) infectious mononucleosis and was discharged with supportive care. However, he was readmitted to the ED a week later due to increased fatigue and shortness of breath. The patient received intravenous steroids and was discharged the same day with the resolution of his symptoms. He came back to the ED two days later, complaining of reduced oral intake and right neck pain. He was found to have enlarged right palatine tonsils with bilateral white exudates. A CT neck showed extensive bilateral cervical lymphadenopathy, mostly on the right than the left side. The patient received one dose of dexamethasone intravenously. His neck pain diminished, and he tolerated oral feeding, so he was discharged.

Four days later, the patient returned to the ED complaining about worsening fatigue and persisting intermittent fevers since the last ED admission. The patient presented tenderness to palpation in his right upper quadrant, splenomegaly, and tender cervical lymph nodes. Laboratory results revealed elevated levels of liver enzymes and ferritin (aspartate aminotransferase 48; alanine aminotransferase 204; alkaline phosphatase of 294; total bilirubin 1.1; and ferritin 1705). The patient was admitted to the wards with the suspicion of EBV hepatitis. Serological tests for hepatitis, rheumatologic diseases, human immunodeficiency virus (HIV), fungal, and viral etiologies were negative. He had a high serum EBV load of 223,000 copies/ml.

The patient received a two-day course of steroids due to pharyngeal edema and cervical lymph node enlargement. The gastroenterology service was consulted due to concern of EBV hepatitis and recommended to stop steroids and monitor liver function tests over the next several days. Seven days following the admission, the laboratory values continued increasing, reaching alkaline phosphatase levels over 600, total bilirubin of 18.8, and ferritin of 5600. Despite this, the patient was asymptomatic and showed clinical improvement during this time, except for tonsillar enlargement on physical examination.

Although we had a clinical suspicion of HLH from admission, during the first six days, the patient did not fit HLH-2004 diagnostic criteria. Extensive infectious, autoimmune, and neoplastic workup was done within the early seven days of admission. His progressive hepatic dysfunction values that were out of proportion were as expected with viral hepatitis and intermittent high fevers; the decision was made to reevaluate for HLH. CT of abdomen and pelvis reported hepatosplenomegaly and reactive retroperitoneal and mesenteric lymphadenopathy. Clinical suspicion for lymphoma was low due to the rapid onset of symptoms, a positive Monospot test, and no other history of weight loss or night sweats. On hospital day seven, a CT chest for the evaluation of continued high-grade fevers showed prominent lower cervical, mediastinal, hilar, and axillary lymph nodes. Multiple spiculated opacities were present throughout all lung lobes, which were interpreted to be a disseminated infectious process. Due to the high EBV load and ferritinemia, a decision was made to start ganciclovir. The liver enzymes and total bilirubin (TBili) levels continued to increase, and with our high suspicion for HLH, IL2-R levels were drawn on day seven. After three days of antiviral therapy, results came back with elevated IL2-R levels (2800 U/ml). Bone marrow biopsy showed trilineage hematopoiesis and megakaryocyte hyperplasia, with many macrophages exhibiting hemophagocytosis.

Due to rapid clinical deterioration, the HLH-94 protocol was started with etoposide and dexamethasone. Rituximab was added due to the high EBV titers and augment clearance. One day later, the patient developed acute kidney, liver failure, and cytopenia. White blood cells dropped from 5000 to 1000 and platelets from 211 to 129. The patient presented multiorgan failure and was transferred to the critical care unit. The clinical course worsened despite treatment and supportive therapy, and he died two days later.

## Discussion

HLH can be triggered by infection, particularly viruses like Epstein-Barr virus, HIV, influenza, and cytomegalovirus. These may be primary infections in otherwise healthy individuals or reactivation in immunosuppression [9]. Cases related to mycobacterium, leishmania, plasmodium, and histoplasmosis have also been described. EBV is the most common viral trigger, seen in around 40% of viral cases, with herpesviruses as a whole accounting for up to 60% of these. HIV is the most common cause in Europe [9]. EBV infection-associated HLH can be diagnosed using polymerase chain reaction (PCR) and serology [8]. It occurs with a substantially higher frequency in pediatric patients but also in adults. Male patients with EBV-associated HLH may have mutations of the SH2D1A gene, suggestive of X-linked lymphoproliferative syndrome (XLPS), and may require screening. XLPS is associated with marked vulnerability to EBV infections, and up to 60% percent of patients with XLPS can go on to develop EBV-HLH [10]. Thrombocytopenia and anemia are identified in almost 80% of adult cases and leukopenia in 69% [11].

Internal organ involvement is frequent and often leads to progressive multiple organ failure such as our patient’s clinical course [12]. It is essential to highlight that prompt diagnosis is critical to avoid poor outcomes. In retrospect, an alternative scenario in our case was that an aggressive lymphoproliferative condition, such as lymphoma, was the driver of HLH. A cervical LN biopsy (from CT-neck findings) could have been done to rule out lymphoma since EBV is associated with 90% of Hodgkin’s lymphoma and 33% of peripheral T-cell lymphoma. However, since the patient met the diagnostic criteria of HLH and was rapidly deteriorating, a decision was made to perform a bone marrow biopsy. Bone marrow is the preferred anatomical site to diagnose suspected hemophagocytic lymphohistiocytosis. Hemophagocytosis is identified in 84% of reported adult cases. While this patient’s HLH progressed quite rapidly and EBV was thought to be the driver, it is essential to point out that lymphoma should also be considered a possible underlying disease in HLH cases. For future cases, an LN biopsy in the right clinical setting should be considered.

The management of HLH focusses on diminishing the inflammation by myelosuppressive and immunosuppressive therapies and treating any triggers [13]. The HLH-94 treatment protocol was designed to handle the inflammatory component of the disease in the pediatric population [14]. The first is accomplished with an eight-week course of etoposide and dexamethasone. This protocol has a 70% response rate. In the case of EBV infection-associated HLH, the HLH-94 protocol remains the mainstay of treatment. Rituximab is often used as a salvage option in these cases, with or without lymphoma [9]. The mechanism of action of rituximab in EBV-associated HLH has been hypothesized to be from the B-cell depletion it induces, as B-cells are a reservoir for EBV [14]. In patients with EBV-related hemophagocytic lymphohistiocytosis with or without associated lymphoma, rituximab is considered a salvage treatment option [15]. Alemtuzumab, cluster of differentiation 52 (CD52)-targeted therapy, is regarded as the second line in case of HLH-94 treatment failure. Secondary HLH, such as the one associated with EBV, does not require further treatment after reaching remission. There is a lack of prospective studies that have investigated the efficacy of these protocols in adults. However, a global analysis of adult case reports shows that therapeutic regimens containing etoposide are associated with better survival in patients with cancer and infection (71%-75%) than in those with autoimmune diseases (57%). Because the mortality rate was 14 times higher in children with EBV-associated hemophagocytic lymphohistiocytosis who were not given etoposide within the first four weeks, it has been suggested that etoposide might act by partly blocking EBV [15].

Allogeneic hematopoietic stem cell transplantation is the current treatment for primary HLH, but it can also have potential applications in adults with secondary HLH who are refractory to treatment [16]. The main predictive factor of response is the administration of intravenous immunoglobulin (IVIG) within two days of the highest ferritin concentrations per some authors. It remains to be seen whether other markers, such as sCD163, are of similar value as serum ferritin for early hemophagocytic syndrome (HS) diagnosis, enabling emergency IVIG treatment [17]. In patients with EBV-related HLH, quantitative analysis of the cell-free EBV genome copy number four months after therapy might help assess the response to treatment and have prognostic value. Even though survival with transplant ranges from 64% to 76% in pediatric studies, there are not enough data to determine the impact of transplantation on survival in adult HLH cases [18].

## Conclusions

EBV infection-associated HLH is a rare disease with high mortality. It is crucial to think about it when facing a patient with fever, cytopenias, hepatosplenomegaly, and hyperferritinemia.
